# A NATIONAL PROFILE OF FRAIL OLDER ADULTS WITH INSUFFICIENT CARE AND MISMATCHED SUPPORT

**DOI:** 10.1093/geroni/igz038.2937

**Published:** 2019-11-08

**Authors:** Jyoti Savla, Karen A Roberto, Laura Sands

**Affiliations:** Virginia Tech, Blacksburg, Virginia, United States

## Abstract

Older adults differ widely both in the care they require and who they rely upon for care. We use data from the National Health and Aging Trends Study (2011; N=3,265; MAge [SD] = 77 [7.74] years, 62% women) to classify community-living older adults based on their care needs and the various informal and formal providers of care. We also examine the type of care they receive, predictors of this care, and its implications on their health. Older adults with a co-residing caregiver were more likely to report that their needs were not being met (OR = 1.67; 95% CI=1.15–2.42), compared to those who received informal care and paid support. Moreover, older adults who needed help with self-care activities, but received help with household activities were more likely to report unmet needs (OR = 1.55; 95% CI=1.13–2.12). Results are discussed in light of sociodemographic factors differences and mismatched support.

